# Evaluation of the stability of heavy metal-containing sediments obtained in the wastewater treatment processes with the use of various precipitating agents

**DOI:** 10.1007/s10661-023-11036-9

**Published:** 2023-03-04

**Authors:** Paweł Lejwoda, Henryk Świnder, Maciej Thomas

**Affiliations:** 1 Department of Environmental Monitoring, Central Mining Institute in Katowice, Plac Gwarków 1, Katowice, Poland; 2 Faculty of Environmental Engineering and Energy, Cracow University of Technology, Kraków, 31-155 Warszawska 24, Poland

**Keywords:** Heavy metals, Galvanic wastewater, Sediments, Saline water, Acid rain, Mobility

## Abstract

The article presents the results of research on the leachability of selected heavy metals (cadmium, nickel, chromium, cobalt, lead, and copper) from solid waste obtained in laboratory processes involved in the industrial treatment of wastewater generated in metal surface treatment plants. The test sludges were precipitated using sodium hydroxide solution, calcium hydroxide suspension, 45% solution sodium trithiocarbonate (Na_2_CS_3_), 15% solution trimercapto-s-triazine, sodium salt (TMT), and 40% solution sodium dimethyldithiocarbamate (DMDTC). The precipitates were treated with artificial acid rain and artificial salt water. After 1, 7, 14, and 21 days of leaching, the concentration of Cd, Co, Cr, Cu, Pb, and Ni in the leachate was determined. Artificial acid rain leached Ni and Cd to a maximum concentration of 724 mg/L and 1821 mg/L, respectively, from the sludge obtained after the application of Na_2_CS_3_, while artificial salt water leached Ni in the maximum amount of 466 mg/L and Cd—max. 1320 mg/L. When Ca(OH)_2_/NaOH was used, the leaching of Cr reached a similar level for both leaching agents, i.e., the maximum for artificial acid rain was 72.2 mg/L and the maximum for artificial salt water 71.8 mg/L. The use of Na_2_CS_3_ or Ca(OH)_2_/NaOH poses a risk of some heavy metals entering the environment, which may have a negative impact on living organisms, whereas the sludges obtained with the use of DMDTC and TMT as precipitants were the most stable under the experimental conditions and did not pose a potential environmental hazard.

## Introduction

With the development of civilization, the increase in consumption, the development of the livestock industry, and the production of goods, the amount of pollution entering the natural environment is increasing. Heavy metals are a very dangerous pollutant, which have a negative impact on human and animal health. The sources of heavy metals include wastewater from various industries and domestic wastewater. This wastewater goes to the wastewater treatment plant, where one of the by-products of the treatment process is sewage sludge, in which heavy metals can accumulate. The use of such sewage sludge for fertilization purposes in agriculture may contribute to the uncontrolled release of heavy metals into, i.e., cereals, vegetables, fruits, and then after consumption to living organisms, contributing to the formation of, e.g., cancer and kidney and liver diseases (Zheng et al., 2022).

Another source of heavy metals causing pollution of surface waters is the metal surface treatment plants, such as etching and electroplating plants, generating a large amount of industrial waste. It poses a significant problem and requires a number of measures to be taken in order to effectively protect the natural environment against its negative impact. In the case of metal surface treatment plants, the main pollutant flux is galvanic wastewater and sludge. They are the source of a number of heavy metals, such as cadmium, chromium, nickel, zinc, lead, and copper. These metals can occur in the form of various inorganic and organic compounds. Subject literature data indicates that untreated industrial wastewater may contain metals in various concentrations, e.g., Cu 59.0 mg/L, Cd 4.50 mg/L, Zn 22.7 mg/L (Thomas et al., 2021), Cu 70.8 mg/L, Sn 3.36 mg/L, Ni 1.1 mg/L (Thomas et al., 2018a, b, c), Cu 49.5 mg/L (Thomas et al., 2018b), 283–36,000 mg/L Cu (copper plating) (Chen et al., 2013; Konstantinos et al., 2011; Seńczuk, 2012), Ni 16.3–547.2 mg/L (nickel plating) (Lekhlif et al., 2014; Peng et al., 2020; Sivaprakash et al., 2015; Varinder & Siby, 2017), and Sn 3100 mg/L (Thomas et al., 2017). During the treatment of wastewater generated in the electrochemical metal treatment processes, a significant amount of solid waste, i.e., galvanic sludge, is produced. Galvanic sludge is considered a hazardous waste due to the content of toxic heavy metals, such as Cr, Ni, Cu, and Zn. In the event that galvanic sludge needs to be stored, these metals pose a significant threat to the fauna and flora present in the natural environment as well as to human health and life. An important factor influencing the safety of galvanic waste storage is their stability and permanent immobilization of heavy metals in the precipitated sludges (Xia et al., 2020). Important factors that have an impact on the permanent immobilization of heavy metal cations in sediments are, among others, the type of precipitant and the conditions of the precipitation process. In order to reduce the environmental risk associated with the storage of this waste, it is necessary to carry out a neutralization process before it is deposited in a landfill.

The developed technologies for reducing the emission of pollutants from electroplating waste deposited in landfills are based on, for example, recycling and recirculation of metal surfaces obtained in the processes of leaching and precipitation of selected metals from the waste to the treatment processes (Świnder & Lejwoda, 2021). These methods allow improving the economic efficiency of the processes carried out, but are frequently associated with the production of secondary pollutants. Therefore, it is thermal treatment and solidification with binding materials that are most often used in the process of stabilization and neutralization of precipitated electroplating waste. The first method is used to a limited extent, only in the case of particularly hazardous types of waste, as its use involves the consumption of significant amounts of energy (Amrane & Bouhidel, 2018; Chou et al., 2011; Huyen et al., 2016). For this reason, currently used methods of neutralizing solid waste generated in galvanic processes are mainly based on its stabilization under storage conditions. The subject literature data indicates a number of methods that consist chiefly in bonding the waste with various binders. One of the most effective and efficient methods is the use of inorganic binders based on cements and fly ash from the power generation industry. These processes utilize the chemical properties of cement materials, both basic (e.g., Portland cement) and synthetic binders, such as zeolites and geopolymers. These binders are most often obtained from fly ash, clay, metakaolin, and blast furnace slag (Álvarez-Ayuso et al., 2008; Hefni et al., 2018). The advantage of using the presented methods of stabilizing and neutralizing waste from electroplating plants is a reduction of carbon dioxide emissions to the atmosphere, an easier preparation process, and lower costs of raw materials for production, energy savings, synthetic binders’ higher resistance to chemical corrosion compared to traditionally obtained cements, and their physicochemical properties (Mehta & Siddique, 2017; Xu et al., 2017).

The research on sludges containing hydroxides, sulfides, and especially trithiocarbonates and dimethyldithiocarbamates of heavy metals from wastewater treatment was carried out to a limited extent. The aim of the investigations was to determine the effect of the type of precipitating agent used in the laboratory treatment of waste from the galvanic metal treatment process on the leachability of selected heavy metals, i.e., cadmium, nickel, copper, cobalt, and chromium, under the conditions of artificial acid rain and salt water prepared in a laboratory. The research is an important supplement to the knowledge on the possibility of storing galvanic waste.

## Material and methods

### Material

The material used in the research was four types of sludge produced in the process of neutralization of acid wastewater from electroplating plants (pH 2.5 ± 0.1, Ni 129 ± 13 mg/L, Co 1.5 ± 0.2 mg/L, Cr 62 ± 6 mg/L, Cu 110 ± 11 mg/L, Pb 59 ± 6 mg/L). Four methods of metal precipitation were applied with the use of 15% NaOH (sample no. 1), 15% TMT (trimercapto-s-triazine, trisodium salt) (sample no. 2), 40% DMDTC (sodium dimethyldithiocarbamate) (sample no. 3), and 45% Na_2_CS_3_ (sodium trithiocarbonate) (sample no. 4) as a precipitating agent. Calcium hydroxide (Ca(OH)_2_, Chempur, Poland), sodium hydroxide (NaOH, analytical grade, Chempur, Poland), TMT (15% solution of trimercapto-s-triazine, trisodium salt, technical grade, Merck, Darmstadt, Germany), DMDTC (40% solution of sodium dimethyldithiocarbamate, technical grade, Chemische Fabrik Wocklum Gebr. Hertin GmbH & Co. KG, Balve, Germany), Na_2_CS_3_, (45% solution of sodium trithiocarbonate, technical grade, Chemiqua, Cracow, Poland), FeCl_3_ (analytical grade, Chempur, Poland), and anionic flocculant (Furoflock CW 277, technical grade, Chemische Fabrik Wocklum GmbH & Co. KG, Germany) were used in the research. The content of heavy metals in the sludge is presented in Table 1. Aqua regia (nitric acid + hydrochloric acid (spectral pure, Merck, Darmstadt, Germany) in a volume ratio of 1:3) was used in the process of wastewater sludge digestion in order to determine the concentration of heavy metals. Additionally, certified multielement solutions for ICP were used (AccuStandard, USA) and high-purity deionized water (conductivity below 0.05 μS/cm, Direct-Q3 UV, Merck Millipore, Burlington, USA). An artificial salt water solution was prepared with the use of NaCl, KCl, KBr, Na_2_SO_4_, NaHCO_3_, H_3_BO_3_, NaF, MgCl_2_‧6H_2_O, CaCl_2_‧6H_2_O, and SrCl_2_‧6H_2_O (analytical grade, Chempur, Poland) according to (Kester et al., 1967). Artificial acid rain was prepared by adding diluted sulfuric acid (analytical grade, Chempur, Poland), to deionized water until reaching pH 4.8 which is the average value of the pH range (4.6–5.0) of 33.3% of precipitation tested in Katowice (Poland) in 2017 (Liana et al., 2018).

**Table 1:** Chemical composition of the sediments

Parameter	Unit	Sample no. 1 Ca(OH)_2_ + NaOH conventional treatment	Sample no. 2 Ca(OH)_2_ + NaOH + TMT	Sample no. 3 Ca(OH)_2_ + NaOH + DMDTC	Sample no. 4 Ca(OH)_2_ + NaOH + Na_2_CS_3_
Co	g/kg	$0.20 \pm 0.03$	$0.15 \pm 0.02$	$0.10 \pm 0.02$	$0.19 \pm 0.03$
Cr	g/kg	$14.0 \pm 2.1$	$13.9 \pm 2.1$	$9.8 \pm 1.5$	$14.7 \pm 2.2$
Cu	g/kg	$31.6 \pm 4.7$	$31.0 \pm 4.7$	$23.6 \pm 3.5$	$39.8 \pm 6.0$
Ni	g/kg	$43.2 \pm 6.5$	$41.3 \pm 6.2$	$30.5 \pm 4.6$	$42.5 \pm 6.4$
Pb	g/kg	$31.3 \pm 4.7$	$33.1 \pm 5.0$	$24.3 \pm 3.6$	$28.2 \pm 4.2$
Cd	g/kg	$17.5 \pm 2.6$	$17.1 \pm 2.6$	$12.2 \pm 1.8$	$18.7 \pm 2.8$

### Research methodology

All experiments were conducted at a constant temperature (20 ± 1 °C), in beakers, each containing 500 ± 5 mL of wastewater, which was mixed with a magnetic stirrer at a speed of 200 rpm (at the stage of metal precipitation) and 50 rpm for 1 min (at the sediment flocculation stage). The tests were carried out in the following way: 15% Ca(OH)_2_ suspension was added to 500 ± 5 mL of wastewater until a pH of 7–7.5 was reached; next, 15% NaOH was dosed to reach a pH of 9–9.5; then, the selected precipitating compound (TMT, DMDTC, and Na_2_CS_3_) was dosed until complete precipitation of heavy metals was achieved, which was confirmed by the drop test. The drop test was performed by adding 25 μL of TMT, DMDTC, or Na_2_CS_3_ to 0.5 mL of the filtered wastewater sample (syringe filter, 0.45 μm).

The absence of sediment or a change in the sample color (negative result) indicated that the dosage of the precipitant was complete; otherwise, dosing was continued until the above test was negative.

In all cases, a slight excess of 1–2% of the precipitation reagents was used. Due to the alkaline properties of TMT, DMDTC, and Na_2_CS_3_ solutions, the pH of the wastewater was corrected with 10% H_2_SO_4_ until the range of 9–9.5 was achieved. Upon completion of precipitation, the precipitates were flocculated by adding 2.0 mL of 0.05% anionic flocculant solution. After 30 min of sedimentation, samples of the treated wastewater were taken from the sediment, filtered through a 0.45 μm membrane filter, and subjected to the tests described in the “Analytical methods” section. The sediments were dewatered using a filter cloth with a grammage of 140–160 g/m^2^ and then dried in the air and in a desiccator at a temperature of 20 ± 1°; next, they were averaged and tested in order to determine their composition and mobility of heavy metals. In order to determine the metal content, the sludge was digested in aqua regia (HNO_3_: HCl, 1:3, v/v), and the resulting solutions were filtered and analyzed by the ICP-OES technique. The aqueous extracts were prepared by weighing the dried and averaged sludge samples obtained at the stage of heavy metal precipitation. 32 aqueous extracts were prepared (4 types of sludge, 4 leaching periods, and 2 leaching agents) and flooded with the leaching agent in a mass ratio (sludge:leaching agent) of 1:10. After each of the leaching periods (i.e., 1 day, 7 days, 14 days, and 21 days), the test solutions were passed through a 0.45 μm syringe filter and, next, analyzed by the ICP-MS technique in order to determine the heavy metal content.

### Analytical methods

The content of metals in the sludges was determined using Inductively Coupled Plasma Optical Emission Spectrometry ICP-OES according to EN ISO 11885:2009 (Optima 5300DV, PerkinElmer, USA) after prior mineralization of the sludges in aqua regia. The content of Cd, Ni, Cu, Cr, Co, and Pb in aqueous solutions after the leaching experiment was determined using inductively coupled plasma mass spectrometry ICP-MS according to EN ISO 17294–2:2016 (NexION 300S, PerkinElmer, USA); the determination of metals in the obtained solutions was performed with the level of uncertainty of 15%, a coverage factor of 2, and a significance level of 95%, and the limit of quantification (LOQ) for each element reached 0.02 mg/L.

## Results and discussion

The efficiency of metal precipitation by the methods described in “Research methodology” was within a range of 98.80–99.94%. The content of individual heavy metals in the sludge obtained with the use of various precipitants is presented in Table 1. The processes taking place during conventional wastewater treatment with Ca(OH)_2_ and NaOH can be described by general reaction Eqs. (1) and (2):

1 $$Me^{2+} + Ca(OH)_2 \to Me(OH)_{2}\downarrow + Ca^{2+}$$

2 $$Me^{2+} + 2NaOH \to Me(OH)_{2}\downarrow + 2Na^{+}$$

The presence of complex compounds in the wastewater may negatively affect the efficiency of the heavy metal precipitation process. In such a case, reagents such as TMT, DMDTC, or Na_2_CS_3_ should be used to improve the efficiency of the process. In the conducted research, the metal removal efficiency in each of the cases reached 98.80–99.94%, which may indicate a low content of complexing compounds. The precipitates obtained after using TMT, DMDTC, or Na_2_CS_3_ are a mixture of the hydroxides of the precipitated metals and the corresponding salts. In the case of Na_2_CS_3_, the produced precipitate is a mixture of sparingly soluble metal hydroxides, trithiocarbonates, and sulfides formed according to the following Eqs. (3)–(5) (Thomas et al., 2021):

3 $$Me^{2+} + 2OH^{-} \to Me(OH)_{2}\downarrow$$

4 $$Me^{2+} + S^{2-} \to MeS\downarrow$$

5 $$Me^{2+}+CS{_3}^{2-}\rightarrow MeCS_3\downarrow$$

**Fig. 1:**
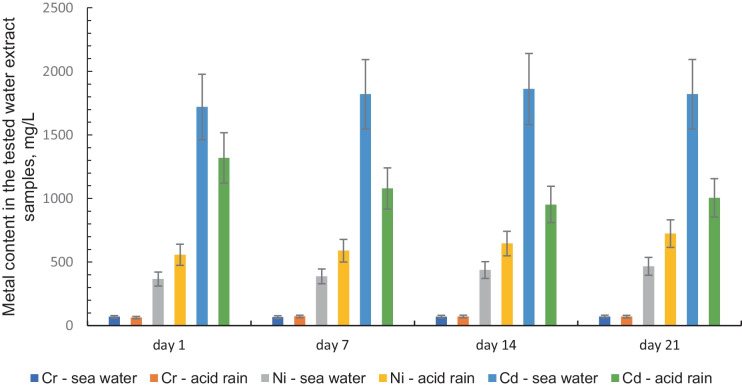
Metal content in the tested water extract samples (for the ratio of sediment mass to water volume reaching 1:10), according to PN-G-11010:1993 under static conditions, at a temp. of 20 ± 1 °C, measurement uncertainty ± 15%

In the next stage of the research, water extracts were prepared and the content of individual metals was determined after 1, 7, 14, and 21 days of the contact between the sludge and water simulating acid rain and water with increased salinity. The results of these investigations have been presented in Table 2 and in Fig. 1.

**Table 2:** Metal content in the tested water extract samples (for the ratio of sediment mass to water volume reaching 1:10, according to PN-G-11010:1993 under static conditions, at a temp. of 20 ± 1 °C)

Sample	Parameter	Leaching agent	Unit	Day 1	Day 7	Day 14	Day 21
Sample no. 1 Ca(OH)_2_ + NaOH conventional treatment	Cr	Sea water	mg/L	$69.1 \pm 10.4$	$66.8 \pm 10.0$	$69.7 \pm 10.5$	$71.8 \pm 10.8$
		Acid rain	mg/L	$63.8 \pm 9.6$	$72.2 \pm 10.8$	$71.8 \pm 10.8$	$70.7 \pm 10.6$
	Ni	Sea water	mg/L	< 0.02	< 0.02	< 0.02	< 0.02
		Acid rain	mg/L	< 0.02	< 0.02	< 0.02	< 0.02
	Cd	Sea water	mg/L	< 0.02	< 0.02	< 0.02	< 0.02
		Acid rain	mg/L	< 0.02	< 0.02	< 0.02	< 0.02
	Co	Sea water	mg/L	< 0.02	< 0.02	< 0.02	< 0.02
		Acid rain	mg/L	< 0.02	< 0.02	< 0.02	< 0.02
	Pb	Sea water	mg/L	< 0.02	< 0.02	< 0.02	< 0.02
		Acid rain	mg/L	< 0.02	< 0.02	< 0.02	< 0.02
	Cu	Sea water	mg/L	< 0.02	< 0.02	< 0.02	< 0.02
		Acid rain	mg/L	< 0.02	< 0.02	< 0.02	< 0.02
Sample no. 4 Ca(OH)_2_ + NaOH + Na_2_CS_3_	Cr	Sea water	mg/L	< 0.02	< 0.02	< 0.02	< 0.02
		Acid rain	mg/L	< 0.02	< 0.02	< 0.02	< 0.02
	Ni	Sea water	mg/L	$367 \pm 55$	$386 \pm 58$	$438 \pm 66$	$466 \pm 70$
		Acid rain	mg/L	$556 \pm 84$	$590 \pm 89$	$646 \pm 97$	$724 \pm 109$
	Cd	Sea water	mg/L	$1720 \pm 258$	$1820 \pm 273$	$1762 \pm 264$	$1821 \pm 273$
		Acid rain	mg/L	$1320 \pm 198$	$1078 \pm 162$	$952 \pm 143$	$1004 \pm 151$
	Co	Sea water	mg/L	< 0.02	< 0.02	< 0.02	< 0.02
		Acid rain	mg/L	< 0.02	< 0.02	< 0.02	< 0.02
	Pb	Sea water	mg/L	< 0.02	< 0.02	< 0.02	< 0.02
		Acid rain	mg/L	< 0.02	< 0.02	< 0.02	< 0.02
	Cu	Sea water	mg/L	< 0.02	< 0.02	< 0.02	< 0.02
		Acid rain	mg/L	< 0.02	< 0.02	< 0.02	< 0.02

Table 2 does not include the results of tests of Sample no. 2 (Ca(OH)_2_ + NaOH + TMT) and sample no. 3 (Ca(OH)_2_ + NaOH + DMDTC), because the concentrations of all the analyzed elements were < 0.02 mg/L.

The conducted chemical analysis of the obtained water extracts has demonstrated that under experimental conditions it is possible to leach out heavy metals from sludge into artificial acid rain and artificial salt water solutions. In the case of using Ca(OH)_2_ and NaOH, followed by Na_2_CS_3_ solutions to precipitate metals from galvanic wastewater, it was found that in the samples of water extracts where artificial salt water was the leaching agent, the concentration of cadmium and nickel reached 1720–1821 mg/L and 367–466 mg/L, respectively, which corresponded to metal leaching within the range of 92.0–97.4% and 8.64–11.0% in terms of a dry sludge weight basis. The experiments in which artificial acid rain was used revealed the presence of cadmium and nickel in the range of 952–1320 mg/L and 556–724 mg/L, which corresponded to metal leaching in the range of 50.9–70.6% and 13.1–17.0% on a dry sludge weight basis. The obtained results may not only indicate the mobility of the tested heavy metals but may also be due to the use of a small (1–2%) excess of the precipitating agent, which could have led to an increase in the solubility of complex compounds (or salts formed in the presence of other substances in the wastewater, e.g., NH_4_^+^) of the CS_3_^2−^ ion with metals, e.g., [Cu(CS_3_)_n_]^n−^, [Ni(CS_3_)_n_]^n−^, and [Cd(CS_3_)_n_]^n−^ (Gattow & Behrendt, 1977; Thomas et al., 2021). In the case of sludge treated with artificial acid rain, the factor that could have a significant impact on the observed phenomenon of metal leaching was the pH of the solution (pH = 4.8). In this case, the digestion of the sludges and the release of metal cations into the aqueous solution could have occurred as a result of the reactions (6)–(8):

6 $$MeCS_{3}\downarrow + 2H_{3}O^{+} \to Me^{2+} + H_{2}CS_{3} + 2H_{2}O$$

7 $$H_{2}CS_{3} \to CS_{2}\uparrow + H_{2}S\uparrow$$

8 $$MeS\downarrow + 2H_{3}O^{+} \to Me^{2+} + H_{2}S\uparrow + 2H_{2}O$$

Chromium was determined in water extracts from wastewater sludges produced due to the use of Ca(OH)_2_ and NaOH as heavy metal precipitants. Both in the case of artificial acid rain and artificial salt water, the chromium content was comparable, reaching 63.8–72.2 mg/L (artificial acid rain) and 66.8–71.8 mg/L (artificial salt water) which corresponded to metal leaching within a range of 4.6–5.2% and 4.8–5.1% on a dry weight basis. The Cr(OH)_3_ precipitate was partially digested in a wide pH range, remaining in equilibrium with the digestion products according to the reaction (9)–(11) (Rai et al., 1987):

9 $$Cr(OH)_{3}\downarrow + 3H_{3}O^{+}\leftrightarrows Cr^{3+} + 6H_{2}O$$

10 $$Cr(OH)_{3}\downarrow + 2H_{3}O^{+} \leftrightarrows CrOH^{2+} + 4H_{2}O$$

11 $$Cr(OH)_{3}\downarrow + H_{3}O^{+} \leftrightarrows Cr(OH)_{2}{^+} + 2H_{2}O$$

## Conclusions

The galvanic sludges produced as a result of applying four precipitants (Ca(OH)_2_/NaOH, TMT, DMDTC, and Na_2_CS_3_) were subjected to leachability tests for 1, 7, 14, and 21 days, using artificial acid rain and artificial salt water as leaching agents in conditions similar to real ones on the basis of which the degree of immobilization of heavy metals in sludges was determined. The analysis of the obtained water extracts did not reveal any leachability of metals (Co, Cu, Ni, Cd, Cr, and Pb) from the sludge produced as a result of using sodium dimethyldithiocarbamate (DMDTC) and trimercapto-s-triazine (TMT) by the available analytical methods. In the case of using NaOH and Na_2_CS_3_, it was demonstrated that some of the examined metals were released from the sludge to the leaching solution. The application of Ca(OH)_2_/NaOH for precipitating metals in the form of hydroxides resulted in the leaching of chromium ions from the sludges in the amount of 63.8–72.2 mg/L under the influence of artificial acid rain and in the amount of 66.8–71.8 mg/L under the influence of artificial salt water, which accounted for, respectively, 4.6% to 5.2% of total chromium contained in the sludge. When Na_2_CS_3_ was used, a release of Cd and Ni ions from the sludges was observed in the amount of 952–1320 mg/L and 556–724 mg/L, respectively, under the influence of artificial acid rain and 1720–1821 mg/L and 367–466 mg/L under the influence of artificial salt water, which accounts for, respectively, 50.9–97.4% of Cd and 8.64%–11.0% of Ni contained in the sludge. A probable release of heavy metal cations from galvanic sludge and consequently a potential leakage to the natural environment disqualify the storage of such sludge without proper protection. The use of TMT or DMDTC allowed to obtain stable precipitates, where heavy metals were not leached out under experimental conditions, which suggests the possibility of safe use of TMT or DMDTC in the process of galvanic wastewater treatment.

## Data Availability

The datasets supporting the conclusions of this article are included within the article.
